# Human papillomavirus E6 alters Toll-like receptor 9 transcripts and chemotherapy responses in breast cancer cells in vitro

**DOI:** 10.1007/s11033-024-10143-1

**Published:** 2024-12-07

**Authors:** Essi Parviainen, Sini Nurmenniemi, Sara Ravaioli, Sara Bravaccini, Aki Manninen, Arja Jukkola, Katri Selander

**Affiliations:** 1 Research Unit of Translational Medicine, University of Oulu, Oulu, Finland; 2 Medical Research Center Oulu, Oulu University Hospital, University of Oulu, Oulu, Finland; 3 IRCCS Istituto Romagnolo per lo Studio dei Tumori (IRST) “Dino Amadori”, Meldola, Italy; 4 Faculty of Medicine and Surgery, “Kore” University of Enna, Enna, Italy; 5 Disease Networks Research Unit, Faculty of Biochemistry and Molecular Medicine, Biocenter Oulu, University of Oulu, Oulu, Finland; 6 Department of Oncology and Radiotherapy, Tampere University Hospital, Tampere, Finland; 7 Faculty of Medicine and Health Technology, Tampere Cancer Center, Tampere University, Tampere, Finland; 8 Department of Oncology and Radiation Therapy, Oulu University Hospital, Oulu, Finland

**Keywords:** Breast cancer, Toll-like receptor 9, Human papillomavirus, Oncoprotein E6, Chemotherapy, Cellular biology

## Abstract

**Background:**

Toll-like receptor 9 (TLR9) is a DNA recognizing receptor expressed also in several cancers. Decreased TLR9 expression is associated with poor prognosis in triple negative breast cancer (TNBC), but the role of TLR9 in breast cancer pathophysiology is currently unclear. Regulation of TLR9 expression in breast cancer is poorly understood. Human papillomavirus (HPV) infections suppress TLR9 expression in cervical cancers but the association between HPV and breast cancer has remained controversial. The aim of this study was to test if HPV16 can suppress TLR9 expression in breast cancer cells and affect cell behavior.

**Methods and results:**

Human T-47D and MDA-MB-231 breast cancer cells were transduced with lentivirus encoding HPV16 E6 oncoprotein. The effects of E6 on TLR9 mRNA and protein expression, and cell proliferation, migration, invasion and sensitivity to chemotherapy were studied in vitro. Breast cancer tissue samples (*n* = 37) were analyzed for the presence of HPV DNA. E6 expression decreased TLR9 mRNA expression in MDA-MB-231 and T-47D cells in hypoxia. E6 expression altered breast cancer cell proliferation and made cells significantly less sensitive to the growth inhibitory effects of chemotherapeutic agents. HPV L1 gene was not detected in a small pilot cohort of clinical breast cancer specimens.

**Conclusion:**

HPV16 may influence breast cancer cell TLR9 transcription and chemotherapy responses and could thereby affect breast cancer prognosis. These results suggest that HPV may have a previously unrecognized role in breast cancer pathophysiology and warrant further studies on the topic.

**Supplementary Information:**

The online version contains supplementary material available at 10.1007/s11033-024-10143-1.

## Introduction

Breast cancer is the most common cancer among women. During the year 2020, over 2.3 million new breast cancer cases and over 680 000 breast cancer deaths were reported worldwide [1]. Despite comprehensive screenings and effective treatments, the incidence is increasing especially in low-resource countries. Gender, aging, and genetic background are the main risk factors. Other intrinsic risk factors are early menarche, late menopause, and high estrogen levels. In addition, lifestyle, such as smoking, alcohol consumption, low physical activity and obesity also increase breast cancer risk [2].

Clinically, breast cancer is divided into three main subtypes based on the expression of estrogen (ER), progesterone (PR) and HER2 receptors [3]. This division is crucial for suitable treatment selection [4]. Nevertheless, despite subtype-specific treatments, such as anti-HER2 and endocrine therapies, the cornerstones of early breast cancer adjuvant treatment have remained the same for decades and continue to include radiation therapy, chemotherapy, and hormonal therapy. Regardless of major improvements in treatment strategies and chemotherapy combinations, approximately 30% of patients that were initially treated in the curative intent for early disease, still die from breast cancer [1]. Thus, further information about the disease pathophysiology remains crucial.

Toll-like receptor 9 (TLR9) is a DNA recognizing receptor belonging to the innate immune system. It is expressed in endosomes and endoplasmic reticulum in several cell types. Bacterial or viral DNA containing unmethylated cytosine–guanine dinucleotide motifs are the main ligands for TLR9, although host-derived DNA can also activate it [5, 6]. Ligand-induced TLR9 stimulation results in a rapid up-regulation of pro-inflammatory cytokines, which eventually activate the adaptive immunity [7]. Since the initial discovery, TLR9 has also been shown to participate in other cellular functions, such as autophagy [8, 9]. TLR9 is widely expressed in various cancers [7]. A recent analysis of a large set of breast cancer samples from The Cancer Genome Atlas revealed that most of the TLRs are significantly downregulated in cancer, as compared with normal epithelium. Also, differences between TLR9 expression and breast cancer subtypes/stages were detected [10]. Low TLR9 expression is associated with poor prognosis specifically in triple negative breast cancer (TNBC) [11, 12]. It is currently unclear whether TLR9 is only a prognostic biomarker, or whether it has a role in breast cancer pathophysiology or treatment responses. Although hypoxia, ERα and testosterone affect TLR9 expression in breast cancer, regulation of TLR9 expression in breast cancer remains poorly understood [13, 14].

Human papillomavirus (HPV) infection is mostly a sexually transmitted disease, which may induce oncogenic transformation in target cells. HPV types 16, 18, 31, 33, 35, 39, 45, 51, 52, 56, 58 and 59 are known to be related to cancer and are therefore classified as high-risk HPVs [15]. Especially the E6 and E7 are thought to be the most salient proteins for the development of malignancies. In addition, *E6* and *E7* are the only HPV genes that remain expressed in HPV-related cancer cells [16]. Association between TLR9 and HPV has been shown in previous studies, where impaired expression and function of TLR9 was observed in cervical [17, 18] and in head and neck cancers [19]. In primary human keratinocytes, HPV16 E6 and E7 proteins were shown to inhibit *TLR9* signaling [20].

Although breast cancer is not typically considered to be HPV-associated, several research groups have demonstrated the presence of HPV DNA in breast cancer tissues. The prevalence of HPV in breast cancers in these small studies have been shown to range from 4 to 42%. In these studies, HPV16 is a commonly detected HPV type [21–25]. However, many studies have concluded that there is no association between HPV and breast cancer [23, 24, 26]. Direct HPV effects on breast cancer have also been studied with cancer cell lines. For example, expression of HPV16 E6 and E7 proteins increased the level of HER2 in breast cancer cells in vitro [27]. Taken together, there is some evidence to suggest that HPV DNA may be incorporated into breast cancer tissues. There are, however, no previous reports on HPV’s effects on TLR9 expression in breast cancer.

## Materials and methods

### Cell culture

Human TNBC MDA-MB-231 (HTB-26) breast cancer cells and the estrogen receptor (ER) expressing T-47D (HTB-133) breast cancer cells were purchased from ATCC (Manassas, Virginia, USA). MDA-MB-231 cells were cultured in Dulbecco’s modified Eagle’s medium supplemented with 10% heat-inactivated fetal bovine serum (Serana Europe, Germany), 2 mM L-glutamine, 100 U/ml penicillin/streptomycin and non-essential amino acids (all obtained from Gibco BRL, Life Technologies, UK). T-47D cells were cultured in RPMI-1640 medium supplemented with 20% heat-inactivated fetal bovine serum, 2 mM L-glutamine, 100 U/ml penicillin/streptomycin, non-essential amino acids and 10 µg/ml insulin (all obtained from Gibco BRL). All cells were cultured in 37 °C, 5% CO_2_, 95% air, except the hypoxia experiments, in which the cells were cultured first in normoxia for 24 h and then transferred to 37 °C, 1% O_2_, 5% CO_2_ (Sci-Tive-N, Ruskinn Technology Ltd, UK). Culture conditions in IncuCyte S3 system (Sartorius, Germany) were 37 °C, 5% CO_2_, 95% air.

### Lentivirus transduction

 Ectopic expression of HPV16 E6 was achieved by transducing cells with dual promoter lentiviral particles containing EF1a promoter driven E6 cDNA and RSV promoter driven GFP-puromycin fusion gene (LVP1136-GP). Lentiviral vectors expressing only the GFP-puromycin fusion gene were used as transduction control (EF1a-Null-GP). Transduction was done according to the manufacturer’s instructions (Gentaur, Belgium). Shortly, 4 × 10^4^ MDA-MB-231 and 1 × 10^5^ T-47D cells were seeded per 24-well. After 24 h, transduction was performed with 50 µl of lentivirus stock (1 × 10^7^ IFU/ml) per well. Lentiviruses including the same promoter as E6 lentivirus but lacking the coding sequence were used as transduction control. After 48 h of transduction, antibiotic selection was performed by supplementing the complete medium of MDA-MB-231 and T-47D cells with 1 µg/ml and 2 µg/ml puromycin (InvivoGen, USA), respectively. After puromycin selection, cells were analyzed using BD FACSAria IIIu flow cytometer with FACS Diva 8.1. software (Becton Dickinson, San Jose, CA). For cell sorting, 25–30% of the cells with the strongest green fluorescent protein (GFP) signal were selected. For MDA-MB-231 cells this selection was performed twice, and for T-47D cells once.

### RNA isolation

MDA-MB-231 and T-47D cells were cultured in 24 h either normoxia or hypoxia. Cells were lysed and RNA was isolated from the cells using NucleoSpin RNA Plus Mini kit according to the manufacturer’s recommendations (Macherey-Nagel, Germany). RNA concentrations were measured with NanoDrop 2000 (Thermo Scientific, USA). DNA residues from RNA samples were removed using DNase I (Thermo Scientific) according to the manufacturer’s instructions.

### RT-qPCR

A total of 1 µg RNA was converted to cDNA using iScript™ Reverse Transcription Supermix for quantitative reverse transcription polymerase chain reaction (RT-qPCR) according to the manufacturer’s instructions (Bio-Rad, USA). The HPV16 E6 detection was performed using custom primers. Custom E6 and housekeeping gene GAPDH primers were purchased from Eurogentec (Belgium) and TLR9 primers from Bio-Rad. Primer sequences are presented in Online Resource 1 (Supplementary Table 1). RT-qPCR was performed using SsoAdvanced Universal SYBR^®^ Green Supermix (Bio-Rad). A standard amplification program (1 cycle of 95 °C for 2 min, 40 cycles of 95 °C for 5 s, 60 °C for 30 s) was used with CFX96 Touch Real-Time PCR Detection System (Bio-Rad). Obtained Ct-values were normalized with GAPDH expression levels for each cDNA and a relative quantification analysis of target cDNA was performed using 2^−ΔΔCt^ method using CFX Maestro 2.2 software (Bio-Rad).

### Western blot analysis

MDA-MB-231 and T-47D cells were cultured in normoxia or hypoxia for 24–96 h, after which cells were harvested using 1 x Cell Lysis Buffer supplemented with 1 mM protease inhibitor (phenylmethanesulfonyl fluoride), both purchased from Cell signaling technology (USA). Concentrations of proteins were measured using Pierce 660 nm Protein Assay (Thermo Scientific™) according to the manufacturer’s instructions. 70 µg of total protein in 1 x Laemmli Sample buffer (Bio-Rad) with 5% β-mercaptoethanol was boiled for 5 min. Samples were electrophoresed into Mini-PROTEAN^®^ TGX™ Precast 4–15% gels (Bio-Rad) and transferred to LF-PVDF membranes (Bio-Rad). Blocking was performed with Intercept Blocking Buffer (LI-COR Biosciences, Germany). Membranes were incubated with TLR9 primary antibody (NBP2-24863, Novus Biologicals, UK) overnight at 4 °C and GAPDH (GeneTex, USA) primary antibody 1.5 h at room temperature. Anti-mouse 800CW and anti-rabbit 680RD antibodies (LI-COR) were used for secondary detection. All antibodies were diluted in 25% blocking buffer and 75% Tris-buffered saline with Tween-20. Protein bands were visualized with Odyssey CLx imaging system (LI-COR Biosciences). The intensities of protein bands were quantified using Image Lab 6.1 (Bio-Rad). GAPDH was used as loading control and normalization of TLR9 protein levels.

### Cell proliferation assays

Cell proliferation was studied by seeding 3.5 × 10^3^ MDA-MB-231 and 2.5 × 10^4^ T-47D cells per 96-well and the growth was imaged using IncuCyte S3 every 3 hours for 2 to 3 days until confluence was reached. Confluency was analyzed using IncuCyte 2020 C software.

### Cell migration assays

Cell migration was analyzed by seeding 1.5 × 10^4^ MDA-MB-231 and 1 × 10^5^ T-47D cells per well (Imagelock 96-well plate, Sartorius, Germany). Before seeding the T-47D cells, the wells were pre-coated with 50 µg/ml collagen type I (rat tail, Corning, USA) dissolved in 0.02 M acetic acid, and allowed to set for 2 h before washing with PBS. After 24 h, scratch wounds were made using WoundMaker (Essen BioScience, USA) as guided in manufacturer’s instructions, and medium was replaced. Cells were imaged every 3 h for 2 to 3 days depending on the cell line using IncuCyte S3 and relative wound density was analyzed using IncuCyte 2020 C software.

### Cell invasion assays

To investigate the effect of E6 transduction on cell invasion, Cultrex UltiMatrix Reduced Growth Factor Basement Membrane Extract (RGF BME) (Bio-Techne, USA) was used in scratch wound assay. Cells were seeded and treated as described in cell migration assays. After wounds were made and medium was replaced, cells were allowed to stabilize in 37 °C for 10 min after which plate was placed on ice for 5 min. Medium was removed and Cultrex RGF BME was added at a protein concentration of 3 mg/ml. After 30 min incubation in 37 °C, Cultrex RGF BME had gelled, and fresh medium was added for the cells. Cells were allowed to invade through the gel and imaged every 3 h for 2 to 3 days using IncuCyte S3. Relative wound density was analyzed using IncuCyte 2020 C software.

### Chemotherapy in vitro

Proliferation of breast cancer cells was studied in the presence of different chemotherapeutic agents, by seeding 2.5 × 10^3^ MDA-MB-231 and 1.5 × 10^4^ T-47D cells per 96-well. Cells were allowed to attach overnight after which medium was replaced with medium containing indicated concentrations of various chemotherapeutic agents. Cells were imaged every 3 hours for 2 to 3 days depending on the cell line using IncuCyte S3. Cell confluency was analyzed using IncuCyte 2020 C software.

### Patient samples

Paraffin-embedded breast cancer specimens (*n* = 37) were collected from breast cancer patients with local or locally advanced disease that were treated at Oulu University Hospital in 2000–2008. The samples were obtained from the biorepository Borealis (Finland). The study was approved by the Regional Medical Research Ethics Committee of the Wellbeing Services County of North Ostrobothnia (EETTMK 50/2021). Written informed consent was obtained from all participants. To demonstrate HPV expression in the specimens, DNA was extracted with AllPrep DNA/RNA/miRNA Universal Kit (Qiagen, Germany) according to the manufacturer’s instructions. Ampliquality HPV-Type Express v3.0 system (AB Analitica, Italy) was used to detect 40 different HPV strains (6, 11, 16, 18, 26, 31, 33, 35, 39, 40, 42, 43, 44, 45, 51, 52, 53, 54, 55, 56, 58, 59, 61, 62, 64, 66, 67, 68 (a, e, b), 69, 70, 71, 72, 73, 81, 82, 83, 84, 87, 89 and 90). Analyses were conducted according to the manufacturer’s instructions.

### Statistical analyses

All experiments have been reproduced independently three times. All results are presented as mean values ± standard error of the mean (SEM). Data was analyzed with the unpaired Student’s t-test using GraphPad Prism v9.3.1 (GraphPad Software Inc, USA). The results were considered significant when p-values were less than 0.05. The figures were prepared using GraphPad Prism.

## Results

### Establishment of stable HPV16 E6 expressing breast cancer cell lines

**Fig. 1:**
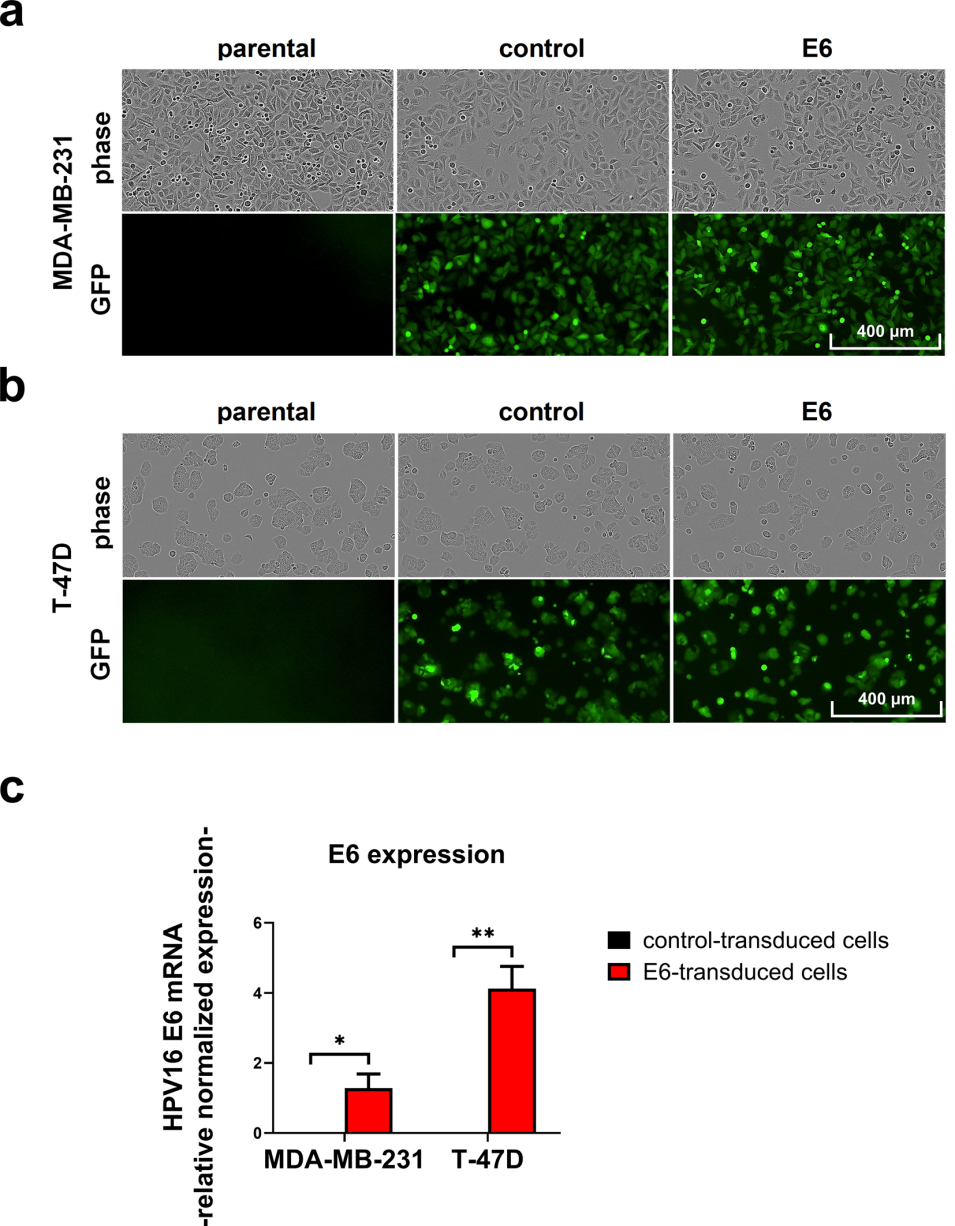
GFP and E6 expression in transduced breast cancer cells. The growth patterns and GFP signaling of MDA-MB-231 (**a**) and T-47D (**b**) cells after antibiotic selection and FACS. Imaging was conducted with IncuCyte S3 (10x). HPV16 E6 expression in E6-transduced cells was verified with qPCR (**c**), in which GAPDH was used for normalization. Results are presented as relative normalized expression (mean ± SEM, *n* = 9, * *p* < 0.05, ** *p* < 0.001)

To study the effect of HPV on TLR9 expression, we transduced parental human MDA-MB-231 and T-47D breast cancer cells with lentiviruses encoding the HPV16 E6 protein. Control cells were established by transducing the cells with corresponding empty vector lentiviruses. Viral transduction did not affect cellular morphology, as the virus-transduced cells appeared similar to the non-transduced parental cells. As expected, only transduced cells exhibited green fluorescence (Fig. 1a-b). Expression of E6 in the E6-transduced cells was confirmed with RT-qPCR (Fig. 1c).

### E6 transduction suppresses TLR9 mRNA expression in TNBC and ER + cells in hypoxia

**Fig. 2:**
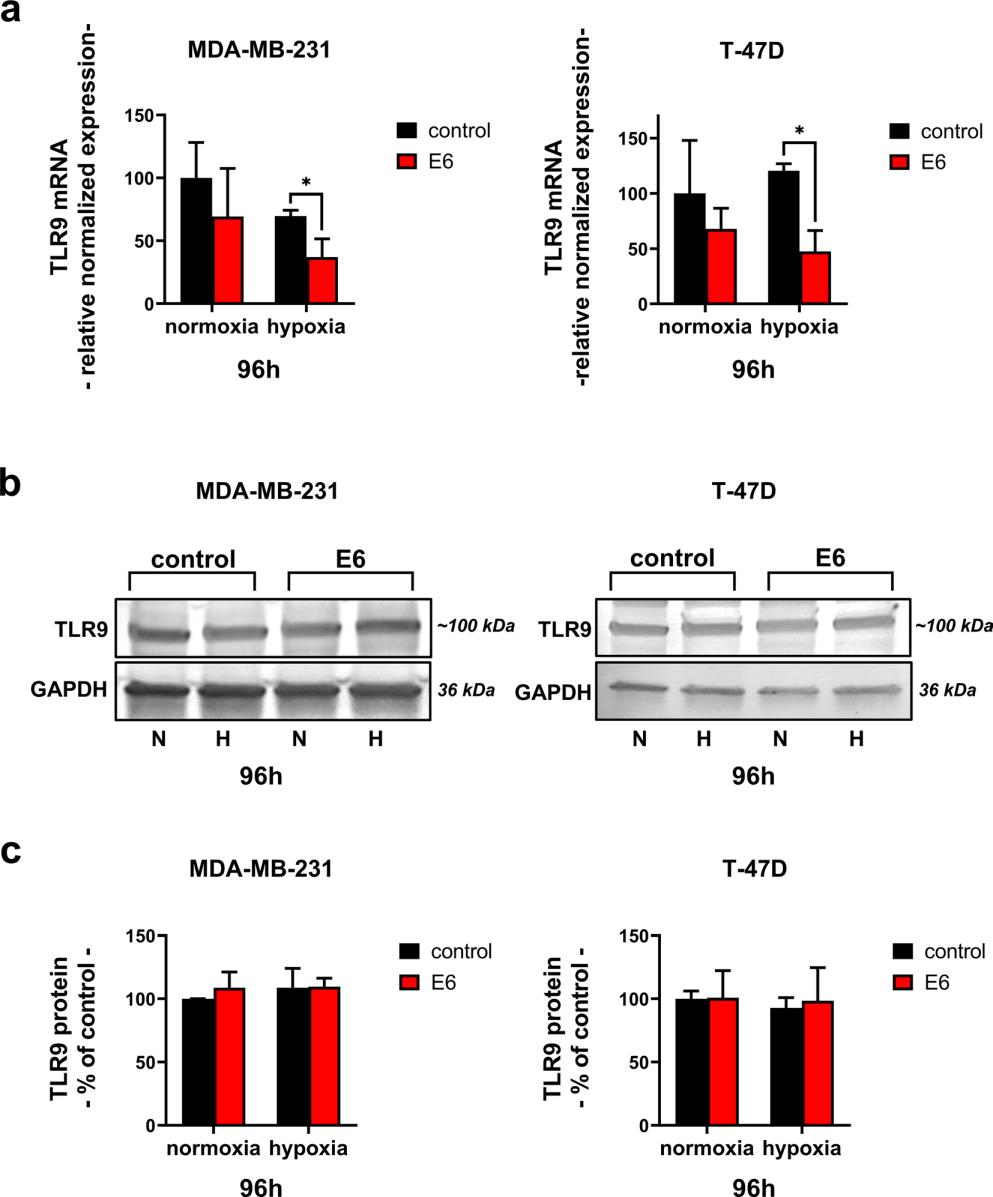
The effect of E6 expression on TLR9 in breast cancer cells. The level of TLR9 mRNA (**a**) and protein (**b-c**) was studied after 96 h of incubation in normoxia and hypoxia in MDA-MB-231 and T-47D cells. GAPDH was used for normalization in both RT-qPCR and Western Blot. Blot images are cropped. Results are presented as mean ± SEM, *n* = 9, * *p* < 0.05

Previous studies have demonstrated that HPV oncoproteins suppress TLR9 expression in cervical cancer epithelial cells [17, 18]. To investigate whether HPV infections similarly affect breast cancer TLR9 expression, MDA-MB-231 and T-47D cells expressing HPV16 E6 and the corresponding control cells were grown both in normoxia and hypoxia. After 24 h of incubation, no significant difference was observed in the expression of TLR9 (Online Resource 1, Supplementary Fig. 1). However, difference at TLR9 mRNA level after 96 h incubation was detected in hypoxia-treated cells (Fig. 2a). Results showed significantly decreased TLR9 mRNA levels in E6-expressing cells compared to control cells. This was seen in both MDA-MB-231 and T-47D cells. No differences were detected in TLR9 protein expression (Fig. 2b-c).

### E6 expression alters breast cancer proliferation, but not migration or invasion in vitro

**Fig. 3:**
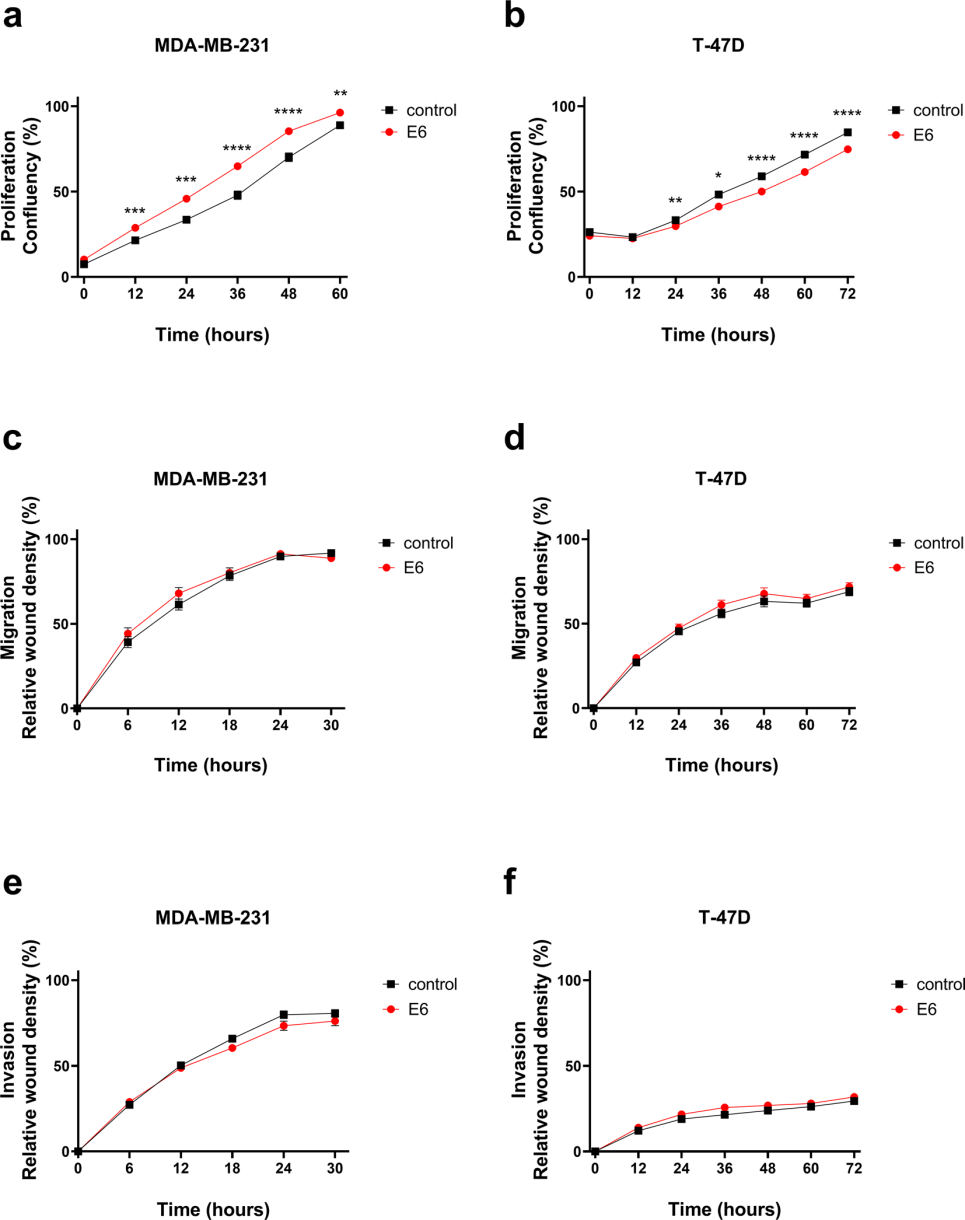
The effect of HPV16 E6 expression on MDA-MB-231 and T-47D breast cancer cell behavior. E6-transduced and control cells were seeded in 96-well plate and proliferation (**a-b**) was monitored with IncuCyte S3. Cell confluency (%) was assessed every 3 h. Migration **(c-d**) and invasion **(e-f**) were studied with scratch wound assay. Relative wound density (%) was measured every 3 h. All results are presented as mean ± SEM, *n* = 18–40, * *p* < 0.05, ** *p* < 0.001, *** *p* < 0.0001, ****p < 0.00001, E6 vs. control cells

To investigate whether HPV16 E6 protein affects breast cancer cell proliferation in vitro, E6-expressing and control cells were seeded onto 96-plates and allowed to grow in normoxia for indicated lengths of time. Proliferation was assessed based on the change in cell confluence (%). E6 expression affected the growth of the two cell lines differently. E6-expressing MDA-MB-231 cells demonstrated a significantly increased proliferation compared to the corresponding control cells (Fig. 3a). The greatest difference was detected at 36 h, when cell confluence was 48% in controls and 65% in E6-expressing cells. In T-47D cells, the effect of E6 expression was opposite, E6-expressing T-47D cells proliferated significantly more slowly than control cells. The largest difference was detected at 66 h, when confluence was 72% in controls and 61% in E6-expressing cells (Fig. 3b).

The effects of E6 expression on breast cancer cell migration were studied with a scratch wound experimental model. MDA-MB-231 cells (Fig. 3c) closed the wound approximately in 27 h. T-47D cells (Fig. 3d) migrated more slowly and 72 h was not sufficient for wound closure. No significant differences in wound closure were observed in either cell line. Representative images of the migration assay are presented in Online Resource 1, Supplementary Fig. 3. The impact of E6 expression on invasive properties of breast cancer cells was studied with scratch wound model, but with the addition of extracellular protein matrix. As shown in Fig. 3, MDA-MB-231 cells (e) closed the wound approximately in 30 h. In the T-47D cells (Fig. 3f) wound closure was slow and the cells practically did not invade at all. No significant difference in invasion between control and E6-expressing cells was observed for either cell line.

### E6 expression alters breast cancer chemotherapy responses in vitro

**Fig. 4:**
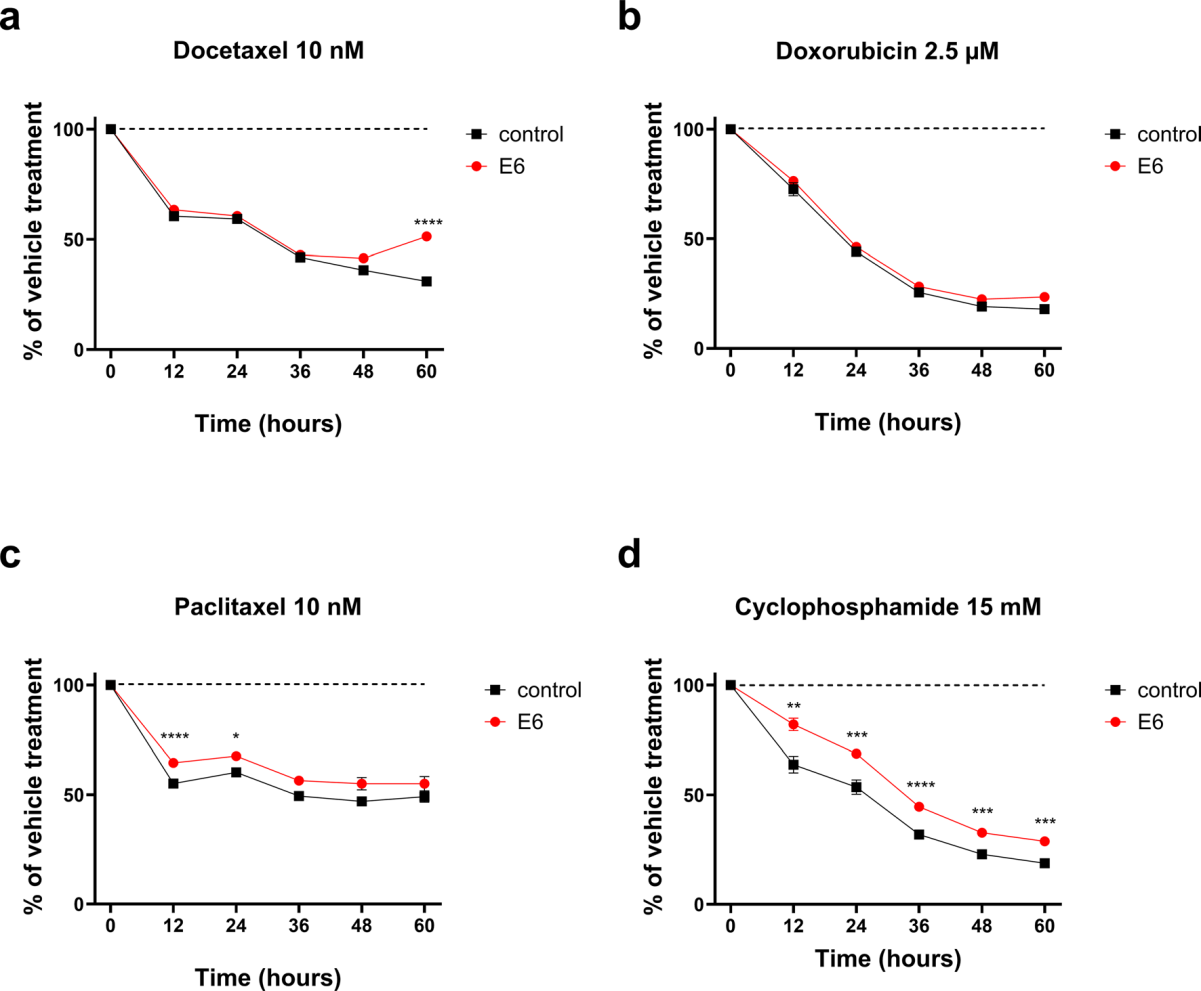
The effect of HPV16 E6 expression on drug sensitivity of TNBC cells. Control and E6-expressing MDA-MB-231 cells were treated with docetaxel (**a**), doxorubicin (**b**), paclitaxel (**c**) and cyclophosphamide (**d**). Cell proliferation was monitored using IncuCyte S3 and images were analyzed with IncuCyte software. Results are presented as % of vehicle control (mean ± SEM, *n* = 18, * *p* < 0.05, ** *p* < 0.001, *** *p* < 0.0001, *p* < 0.00001, E6 vs. control cells)

**Fig. 5:**
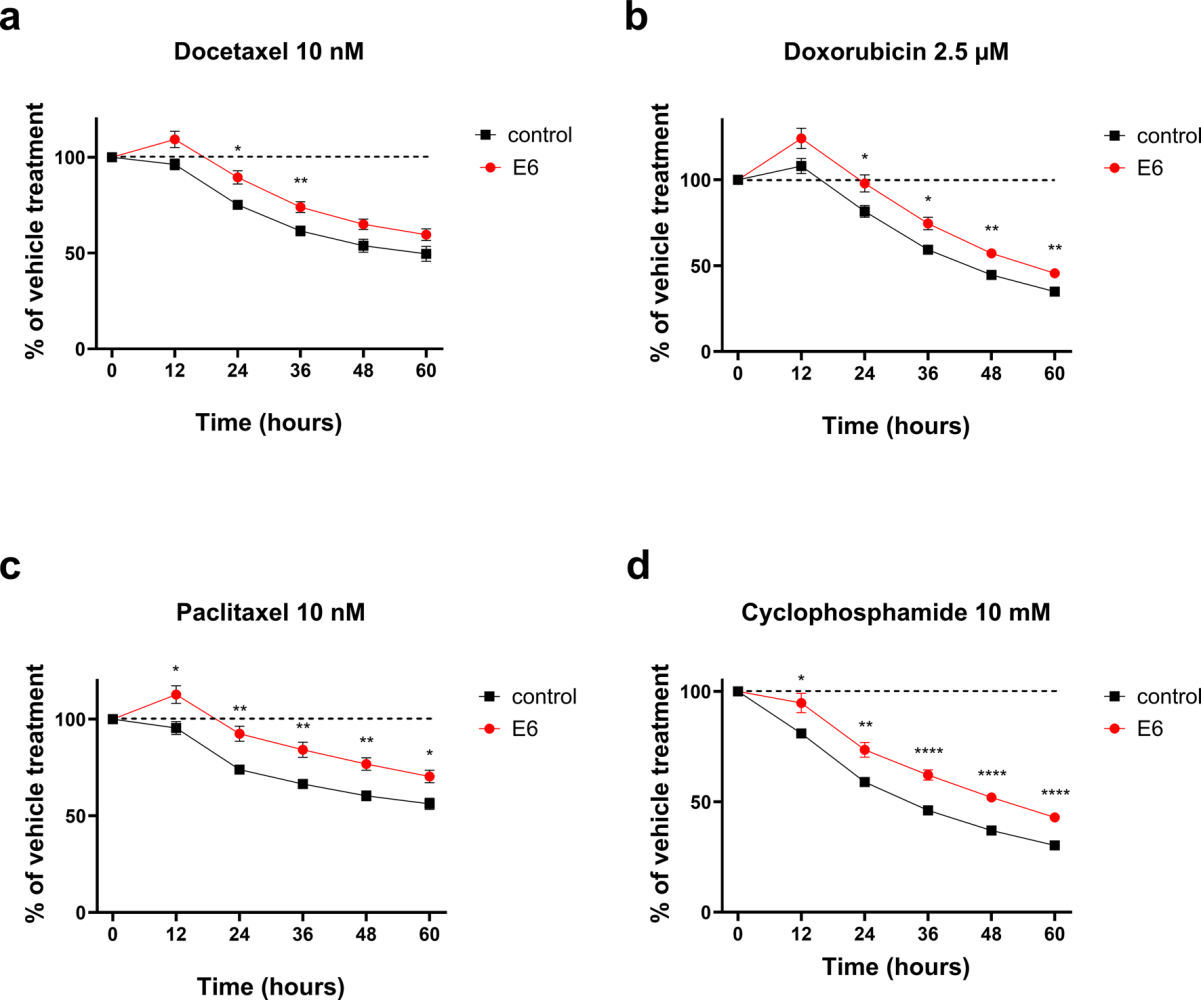
The effect of HPV16 E6 expression on drug sensitivity of ER + breast cancer cells. Control and E6-expressing T-47D cells were treated with docetaxel (**a**), doxorubicin (**b**), paclitaxel (**c**) and cyclophosphamide (**d**). Cell proliferation was monitored using IncuCyte S3 and images were analyzed with IncuCyte software. Results are presented as % of vehicle control (mean ± SEM, *n* = 18, * *p* < 0.05, ** *p* < 0.001, *** *p* < 0.0001, **** *p* < 0.00001, E6 vs. control cells)

We next investigated whether HPV16 E6 expression affects chemotherapy responses in these cells. The chemotherapeutic agents were chosen to represent those used in early breast cancer, in the adjuvant setting. The drug concentrations were selected to mirror those detected in patient’s plasma [28–31]. The cells were grown in the presence of the indicated chemotherapeutic agents, for indicated lengths of time. Cell proliferation was analyzed with Incucyte S3, based on cell confluency. Both E6-transduced cell lines demonstrated significantly decreased sensitivity to the growth inhibitory effects of chemotherapeutic agents. In MDA-MB-231 cells the effect was most prominent with cyclophosphamide (Fig. 4). In T-47D cells the effect was seen with all tested chemotherapeutic agents (Fig. 5).

### HPV DNA detection in clinical breast cancer specimens

Finally, we investigated the presence of HPV L1 gene in DNA samples obtained from a small cohort of local breast cancer samples. We used a commercially available kit including single-step PCR and reverse line blot detecting 40 different HPV genotypes. With this approach, no HPV DNA was detected. The results are presented in Online Resource 1 (Supplementary Fig. 2).

## Discussion

TLR9 is an innate immune system DNA receptor, which is widely expressed in breast and other cancers [11, 12, 32, 33]. Low TLR9 expression in breast cancer tissue is associated with poor prognosis, specifically in TNBC [11, 12]. It is known that hypoxia, ERα and testosterone affect TLR9 expression in breast cancer cells [11, 14, 34]. However, regulation of TLR9 expression in breast cancer is only superficially understood. HPV regulates TLR9 expression in other cancers, such as cervical [17, 18], and head and neck [19] cancers. Although the role of HPV in breast cancer is highly controversial, several research groups have shown that HPV may be present in breast cancer [22, 25]. The aim of this study was therefore to investigate whether HPV infections could also regulate TLR9 expression also in breast cancer.

We show here that HPV16 E6 does regulate TLR9 transcripts also in breast cancer cells. The effect was best seen at the mRNA level and after 96 h incubation in hypoxia. Hypoxia was included in these studies, as the cancer microenvironment becomes hypoxic upon tumor progression, and hypoxia regulates TLR9 expression in breast cancer [11, 35]. No effects on protein expression were detected, except in TNBC cells after a 24 h culture period in normoxia. Our results further demonstrate that hypoxia itself may suppress TLR9 expression, at least at the mRNA level. This result is opposite to our previous publications, which demonstrated that hypoxia increased TLR9 mRNA and protein levels [11, 14]. The difference between the experimental settings of these studies is the level of hypoxia (1% vs. 5% O_2_). Conditions used for the current experiments (1% O_2_) surprisingly demonstrated TLR9 mRNA suppression, and lower O_2_ level might actually better mimic the conditions in the breast cancer microenvironment [36, 37]. Thus, hypoxia alone may be one explanation for low TLR9 expression levels in some breast cancers.

HPV16 E6 also affected breast cancer proliferation, but the effects were the opposite between TNBC and ER + cells; E6 increased the proliferation of TNBC MDA-MB-231 and decreased it in ER+ T-47D breast cancer cells. The reason for the opposite effect of E6 in different breast cancer cell lines may depend on their estrogen receptor expression profiles. Interestingly, in HPV-related head and neck squamous cell carcinomas, higher ERα expression was found to associate with improved survival, and estrogen was found to downregulate the expression of E6 and E7 by repressing the viral long control region of HPV16 [38]. In addition, according to Kano and colleagues, ERα expression was observed as a favorable prognostic factor in HPV-positive oropharyngeal cancer [39]. The underlying mechanism could be that an apolipoprotein B mRNA editing enzyme, catalytic polypeptide-like 3 (APOBEC3), induced by estrogen, is able to induce double-strand breaks of DNA leading to cell death [40].

Interestingly E6-infected breast cancer cells appeared less sensitive to the growth inhibitory effects of standard chemotherapeutic agents used especially in early breast cancer. This effect was seen especially in ER+ T-47D cells, and with cyclophosphamide also in TNBC MDA-MB-231 cells. This finding requires further characterization and needs to be confirmed in vivo. In general, our finding stands in contrast with what is known about chemosensitivity about typical HPV-positive cancers [41]. Nevertheless, in hypopharyngeal squamous cell carcinoma (HSCC), chemotherapy only improved survival outcomes in HPV-negative cancers, but not in HPV-positive, raising the question of chemosensitivity also in HPV+ HSCC cancers [42].

Finally, one aim of this work was also to study the presence of HPV in breast cancer specimens in a small cohort from Northern Finland. The results were negative for all studied HPV subtypes. To evaluate the presence of HPV in breast cancer more reliably, the experiment should be repeated in a large number of samples covering several different breast cancer subtypes. Currently, there are no standardized methods to detect HPV from cancer tissue samples. Available methods and test kits are detecting different parts of HPV; DNA, RNA, oncoproteins, and specific serum antibodies. In the progression of HPV-related cancer, E6 and E7 are thought to be only genes that remain expressed in HPV-related cancer cells [16]. Many studies, including ours, used PCR protocol to detect HPVs late gene 1 (L1) from breast cancer samples [43–45]. L1 gene encodes major capsid protein 1, which is needed for viral assembly and maturation [46]. However, some studies of cervical and anal cancers have demonstrated that L1 expression is lost during the viral integration [47, 48]. This raises the question of how sensitive L1-based HPV detection methods are for screening cancer samples for possible old HPV infection.

In conclusion, these results suggest that HPV16 infections may regulate TLR9 transcripts in breast cancer cells. The outcomes of such infection may be influenced by the ER status of the cells and the oxygen level of the cancer microenvironment. Our results further suggest that if HPV16 indeed infects breast cancer cells, it may change the chemo-responsiveness of the cells and thereby, affect breast cancer outcomes. These issues need to be investigated with large clinical cohorts, and in pre-clinical models.

## Electronic supplementary material

Below is the link to the electronic supplementary material.

Supplementary Material 1

## Data Availability

The authors declare that the data supporting the findings of this study are available within the paper or supplementary information. The individual datasets generated and/or analyzed during the current study are available from the corresponding author on reasonable request.

## Abbreviations

TLR9: Toll-like receptor 9
HPV: Human papillomavirus
mRNA: Messenger RNA
E6: Early protein 6
L1: Human papillomavirus major capsid protein
ER: Estrogen receptor
PR: Progesterone receptor
HER2: Human epidermal growth factor receptor 2
TNBC: Triple negative breast cancer
GAPDH: Glyceraldehyde-3-phosphate dehydrogenase
GFP: Green fluorescent protein
HSCC: Hypopharyngeal squamous cell carcinoma
SEM: Standard error of the mean
