# Supplementary material for: Human papillomavirus E6 alters Toll-like receptor 9 transcripts and chemotherapy responses in breast cancer cells in vitro
Source: Mol Biol Rep. 2024 Dec 7;52(1):43. doi: 10.1007/s11033-024-10143-1 (PMC11625066; doi:10.1007/s11033-024-10143-1)
Supplement: Supplementary file 1 — Supplementary Material 1 [file 11033_2024_10143_MOESM1_ESM.docx]

Human papillomavirus E6 alters Toll-like receptor 9 transcripts and chemotherapy responses in breast cancer cells *in vitro*

Molecular Biology Reports

Essi Parviainen, Sini Nurmenniemi, Sara Ravaioli, Sara Bravaccini, Aki Manninen, Arja Jukkola, Katri Selander

Correspondence:

Sini Nurmenniemi

Research Unit of Translational Medicine, University of Oulu, Oulu, Finland

Medical Research Center Oulu, Oulu University Hospital, University of Oulu, Oulu, Finland

[sini.nurmenniemi@oulu.fi](mailto:sini.nurmenniemi@oulu.fi)

**Supplementary Information**

| **Gene** | **Forward 5’ – 3’** | **Reverse 5’ – 3’** | **Manufacturer** |
| --- | --- | --- | --- |
| **E6 (HPV16)** | ATG CAC CAA AAG AGA ACT GCA | CAG CTG GGT TTC TCT ACG TG | Eurogentec |
| **GAPDH** | ACC ACA GTC CAT GCC ATC AC | TCC ACC ACC CTG TTG CTG TA | Eurogentec |
| **TLR9** | (Amplicon Context Sequence)  CCT ACA TCC CAT GAG GGC CTC ACA CCT GTC CTC TAC CAA GCC CAG GGA GGA GCT AAG GCC CAG AGC TCA GGC AGA GAG CAG GGA GAG ATG GGA ATT CTG GAT AGC ACC AGT AGC GGG TAC ACC TTG CT | | Bio-Rad |

**Supplementary table 1**. Primer sequences for RT-qPCR.


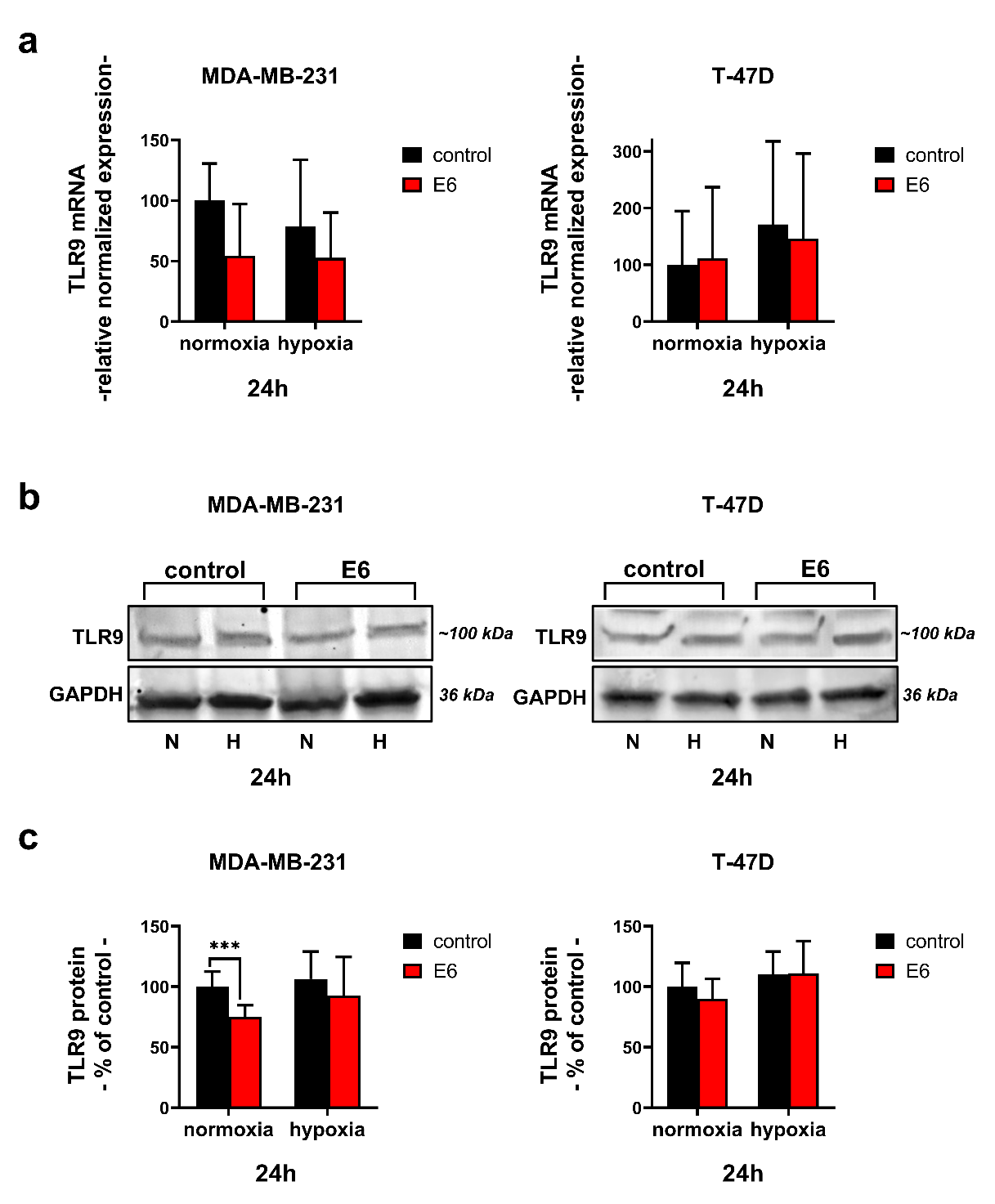


**Supplementary figure 1.** The effect of E6 expression on TLR9 mRNA **(a)** and protein **(b-c)** expression in breast cancer cells. The effect was studied after 24 h incubation in normoxia and hypoxia in MDA-MB-231 and T-47D cells. GAPDH was used for normalization in both RT-qPCR and Western Blot. Blot images are cropped. Results are presented as mean ± SEM, n=9, *** p<0.001


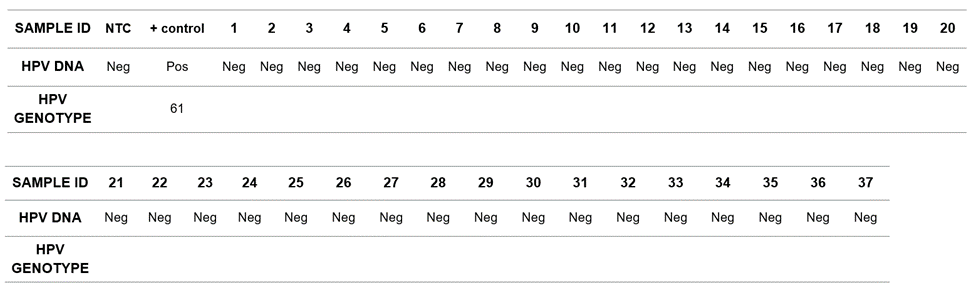


**Supplementary figure 2.** HPV DNA detection from clinical breast cancer samples**.** DNA from clinical breast cancer samples was extracted, and the HPV genotyped with single-step PCR and reverse line blot. Negative control with no template (NTC) was found negative for HPV and positive control showed HPV positivity for genotype 61. All tissue samples (n=37) were found negative for HPV.


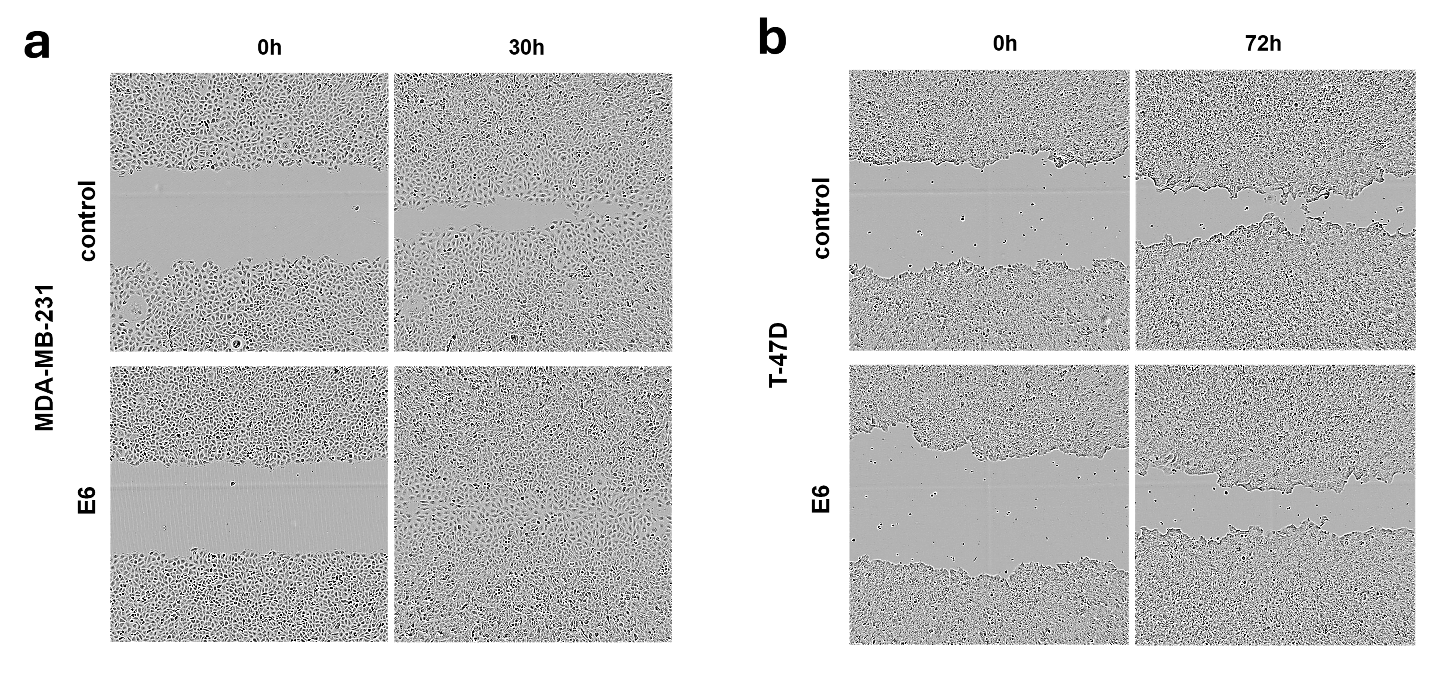


**Supplementary figure 3.** Representative images of the scratch wounds from the migration assays of MDA-MB-231 (**a**) and T-47D (**b**) cells. Wound closure was observed using IncuCyte S3 (20x).
